# 117 The Development of a Patient-centered, Evidence-based and Theoretically-informed Physical Activity Behaviour Change Intervention for Young People who have had Cancer

**DOI:** 10.1093/eurpub/ckae114.111

**Published:** 2024-09-26

**Authors:** Jennifer Fitzpatrick, Kieran Dowd, Niall Moyna, Cliona Godwin, Mairéad Cantwell

**Affiliations:** Technological University of the Shannon, Athlone, Ireland; Irish Cancer Society, Ireland; SHE Research Group, Athlone, Ireland; Technological University of the Shannon, Athlone, Ireland; SHE Research Group, Athlone, Ireland; Dublin City University, Ireland; Children’s Health Ireland at Crumlin, Ireland; Technological University of the Shannon, Athlone, Ireland; SHE Research Group, Athlone, Ireland

## Abstract

**Purpose:**

The purpose of this study was to describe the development of CHAMPs, the CHildhood and adolescent cancer survivors’ physical Activity (PA) and Movement Programme. CHAMPs aims to increase participants’ PA levels, physical function and quality of life.

**Methods:**

Programme development was guided by the Medical Research Council’s Framework for the development of complex interventions and The Behaviour Change Wheel. A review of literature was conducted to identify i) determinants of PA behaviour, maintenance and adherence, and ii) previous successful PA interventions for this population. Additionally, 31 interviews were conducted, which sought to identify important components for inclusion within a PA intervention, from the perspectives of young people who have had cancer (aged 12-25 years), their parents and healthcare professionals. A workshop was conducted with key stakeholders to determine intervention acceptability, including programme appeal and engagement, and to obtain recommendations for improvements.

**Results:**

Recommendations generated from the literature review were summarised in statements of findings (n = 26) which informed programme development, and included the need for a personalised PA intervention which is underpinned by behavioural theory and incorporates multi-modal PA (e.g. strength, flexibility, endurance PA). Ten recommendations were generated from interviews, which emphasised the importance of face-to-face PA sessions and the inclusion of educational components. Stakeholder workshop recommendations (n = 10) included strategies to optimise engagement, including shorter session length for educational components and motivational interviewing (MI) workshops with no parent present to optimise participant engagement. All of the recommendations obtained were synthesized and produced CHAMPs, a 12-week multi-component PA intervention. CHAMPs includes: i) 12 personalised PA sessions (n = 8 home-based; 4=online), ii) 3 MI workshops, iii) 3 educational workshops, iv) PA equipment (e.g. beanbags, hoops) and v) PA information booklet.

**Conclusions:**

This study is the first to develop a PA behaviour change intervention for young people who have had cancer using this systematic approach which has produced a novel patient-centred, evidence-based and theoretically-informed PA programme. CHAMPs will be subsequently tested in a feasibility study.

**Funding Source:**

This work is co-funded by the Irish Cancer Society Social, Nursing, Allied Health Research Scholarship CRS21FIT, and The Technological University of the Shannon President’s Doctoral Scholarship.

